# Bariatric surgery: an IDF statement for obese Type 2 diabetes

**DOI:** 10.1111/j.1464-5491.2011.03306.x

**Published:** 2011-06

**Authors:** J B Dixon, P Zimmet, K G Alberti, F Rubino, on behalf of the International Diabetes Federation Taskforce on Epidemiology and Prevention

**Affiliations:** * Baker IDI Heart and Diabetes InstituteMelbourne, Victoria, Australia; † Imperial College LondonLondon, UK; ‡ Weill Cornell Medical College of Cornell UniversityNew York, NY, USA

## Abstract

The International Diabetes Federation Taskforce on Epidemiology and Prevention of Diabetes convened a consensus working group of diabetologists, endocrinologists, surgeons and public health experts to review the appropriate role of surgery and other gastrointestinal interventions in the treatment and prevention of Type 2 diabetes. The specific goals were: to develop practical recommendations for clinicians on patient selection; to identify barriers to surgical access and suggest interventions for health policy changes that ensure equitable access to surgery when indicated; and to identify priorities for research. Bariatric surgery can significantly improve glycaemic control in severely obese patients with Type 2 diabetes. It is an effective, safe and cost-effective therapy for obese Type 2 diabetes. Surgery can be considered an appropriate treatment for people with Type 2 diabetes and obesity not achieving recommended treatment targets with medical therapies, especially in the presence of other major co-morbidities. The procedures must be performed within accepted guidelines and require appropriate multidisciplinary assessment for the procedure, comprehensive patient education and ongoing care, as well as safe and standardized surgical procedures. National guidelines for bariatric surgery need to be developed for people with Type 2 diabetes and a BMI of 35 kg/m^2^ or more.

## Review criteria

The working group reviewed literature focusing on bariatric surgery published between 1991 and 2010, in the areas of national and international guidelines, systematic reviews of the literature and high-quality clinical trials for the treatment of obesity and diabetes in adults and adolescents. The group synthesized the available evidence for efficacy, safety and cost-effectiveness of the established bariatric procedures in relation to current standard therapy for people with obesity and Type 2 diabetes. The group also explored weight loss and non-weight loss effects of the surgery on glycaemic control and novel gastrointestinal procedures and devices being developed to treat Type 2 diabetes. All papers identified were English-language, full-text papers.

## Executive Summary

### Text Box 1: Background

**Table:** 

• Obesity and Type 2 diabetes are serious chronic diseases associated with complex metabolic dysfunctions that increase the risk for morbidity and mortality.
• The dramatic rise in the prevalence of obesity and diabetes has become a major global public health issue and demands urgent attention from governments, healthcare systems and the medical community.
• Continuing population-based efforts are essential to prevent the onset of obesity and Type 2 diabetes. At the same time, effective treatment must also be available for people who have developed Type 2 diabetes.
• Faced with the escalating global diabetes crisis, healthcare providers require as potent an armamentarium of therapeutic interventions as possible.
• In addition to behavioural and medical approaches, various types of surgery on the gastrointestinal tract, originally developed to treat morbid obesity (‘bariatric surgery’), constitute powerful options to ameliorate diabetes in severely obese patients, often normalizing blood glucose levels, reducing or avoiding the need for medications and providing a potentially cost-effective approach to treating the disease.

### Text box 2: Bariatric surgery

**Table:** 

• Bariatric surgery is an appropriate treatment for people with Type 2 diabetes and obesity not achieving recommended treatment targets with medical therapies, especially when there are other major co-morbidities.
• Surgery should be an accepted option in people who have Type 2 diabetes and a BMI of 35 kg/m^2^ or more.
• Surgery should be considered as an alternative treatment option in patients with a BMI between 30 and 35 kg/m^2^ when diabetes cannot be adequately controlled by optimal medical regimen, especially in the presence of other major cardiovascular disease risk factors.
• In Asian, and some other ethnicities of increased risk, BMI action points may be reduced by 2.5 kg/m^2^.
• Clinically severe obesity is a complex and chronic medical condition. Societal prejudices about severe obesity, which also exist within the healthcare system, should not act as a barrier to the provision of clinically effective and cost-effective treatment options.
• Strategies to prioritize access to surgery may be required to ensure that the procedures are available to those most likely to benefit.
• Available evidence indicates that bariatric surgery for obese patients with Type 2 diabetes is cost-effective.
• Bariatric surgery for Type 2 diabetes must be performed within accepted international and national guidelines. This requires appropriate assessment for the procedure and comprehensive and ongoing multidisciplinary care, patient education, follow-up and clinical audit, as well as safe and effective surgical procedures. National guidelines for bariatric surgery in people with Type 2 diabetes and a BMI of 35 kg/m^2^ or more need to be developed and promulgated.
• The morbidity and mortality associated with bariatric surgery is generally low and similar to that of well-accepted procedures such as elective gall bladder or gallstone surgery.
• Bariatric surgery in severely obese patients with Type 2 diabetes has a range of health benefits, including a reduction in all-cause mortality.
• A national registry of persons who have undergone bariatric surgery should be established in order to ensure quality patient care and to monitor both short- and long-term outcomes.
• In order to optimize the future use of bariatric surgery as a therapeutic modality for Type 2 diabetes, further research is required.

## Introduction

### Why is this position statement needed?

The global prevalence of Type 2 diabetes is rising dramatically, driven by an ‘obesogenic’ environment that favours increasing sedentary behaviour and easier access to attractive calorie-dense foods acting on susceptible genotypes [1]. The most recent global predictions by the International Diabetes Federation (IDF) suggest that there are 285 million people with diabetes currently worldwide. This is set to escalate to 438 million by 2030 [2], with a further half billion at high risk. Diabetes is looming as one of the greatest public health threats of the 21st century.

Type 2 diabetes is a risk factor for vascular damage: both microvascular (retinopathy; nephropathy and neuropathy) and macrovascular (premature and more extensive cardio-, cerebro- and peripheral vascular disease). Premature mortality and morbidity in diabetes result from such complications. The disease results from inadequate insulin production and action and results in hyperglycaemia, but is also associated with multiple other dysfunctions involving lipid metabolism, oxidative stress, inflammation and haemato-rheology. In addition, obesity, by itself, generates similar cardio-metabolic dysfunction [3].

The dramatic rise in the prevalence of obesity and diabetes has become a major global public health issue [2]. The problem is complex [4] and will require strategies at many levels to prevent, control and manage.

There is increasing evidence that the health of obese people with Type 2 diabetes, including the metabolic control of diabetes and its associated risk factors, can benefit substantially from bariatric surgery—that is, surgical procedures to produce substantial weight loss [5,6].

Several gastrointestinal (GI) operations that were originally designed to treat morbid obesity also cause dramatic improvement of Type 2 diabetes and can effectively prevent progression from impaired glucose tolerance to diabetes in severely obese individuals [7]. In addition, bariatric surgery has been shown to substantially improve hypertension, dyslipidaemia and sleep apnoea [8] and several reports have documented an improvement of overall survival [5] and specific reduction in diabetes-related mortality [9].

Despite a number of evidence-based reviews and consensus statements having been published regarding the utilization of bariatric surgery in patients with obesity and diabetes [10–15], the IDF has not previously considered this rapidly developing area of care for worldwide use. Therefore, a need exists for worldwide expert guidance in the preoperative evaluation, choice of interventional procedure, perioperative management and long-term care of patients who seek surgery to improve diabetes control.

The IDF Taskforce on Epidemiology and Prevention convened a consensus working group of diabetologists, endocrinologists, surgeons and public health experts in December 2010 to discuss the appropriate role of bariatric surgery and other gastrointestinal interventions in the treatment and prevention of obesity and Type 2 diabetes. The specific goals of the panel were: to develop practical recommendations for clinicians; to identify barriers that currently prevent access to surgery and suggest interventions for health policy changes that ensure equitable access to surgery when indicated; and to identify priorities for clinical research.

This consensus statement considers primarily established bariatric surgical procedures. It is acknowledged that this is an emerging field and there is a large range of novel extraluminal and endoluminal gastrointestinal surgical procedures and devices that are in the development phase. Some focus primarily on weight loss and others additional non-weight loss metabolic benefits. The use of these requires further validation before they can be recommended.

### How is obesity defined?

Obesity is usually classified by body mass index (BMI), calculated as body weight in kilograms divided by the height in metres squared (kg/m^2^). Classifications of BMI, as defined by the World Health Organization (WHO), based on associations with adverse health consequences, are listed in Table 1. Other methods, including waist circumference and central and peripheral fat mass, have also been used, but currently the clearest evidence suggests continued use of BMI as an index of obesity, particularly when BMI exceeds 30 kg/m^2^.

**Table 1:** The classification of weight category by BMI

	BMI (kg/m^2^)	
	
Classification	Principal cut-off points	Cut-off points for Asians*
Normal range	18.5–24.9	18.5–22.9
		23.0–24.9
Pre-obese	25.0–29.9	25.0–27.4
		27.5–29.9
Obese class I	30.0–34.9	30.0–32.4
		32.5–34.9
Obese class II	35.0–39.9	35.0–37.4
		37.5–39.9
Obese class III	≥ 40.0	≥ 40.0

* For Asian populations, classifications remain the same as the international classification, but that public health action points for interventions are set at 23, 27.5, 32.5 and 37.5 kg/m^2^ [74].

We address eligibility and prioritization for bariatric surgery within the coloured zones above.

Source: Adapted from the World Health Organization (WHO) 2004 [75].

BMI categories have been developed primarily in populations of mainly European ethnicity, and often underestimate health risks in other populations. In addition, BMI does not necessarily reflect the proportion of body weight that consists of fat or the distribution of fat: both these aspects of body composition can affect the health risks of excess weight. Nevertheless, at present, in the absence of a better alternative, BMI is the internationally accepted standard used by researchers and policymakers to allocate individuals to different size categories.

Clinically severe or ‘morbid’ obesity is considered to be class III obesity or class II obesity with significant obesity-related co-morbidity, including Type 2 diabetes (Table 1). Additional cut-points for public health action have been suggested to address the increased risk of diabetes and cardiovascular disease in Asian populations and further investigation should examine other at-risk ethnicities.

### The link between obesity and Type 2 diabetes

Type 2 diabetes is a heterogeneous disorder and, while its causes have yet to be fully explained, obesity is considered the primary risk factor [16]. It has been estimated that the risk of developing Type 2 diabetes is increased 93-fold in women and 42-fold in men who are severely obese rather than of healthy weight [17,18]. A small proportion of people with Type 2 diabetes, approximately 15% in populations of European origin, are not overweight [19].

In the short term, even modest weight loss in people with Type 2 diabetes who are overweight or obese is associated with improvements in glycaemic control and associated conditions such as hypertension and dyslipidaemia [20]. However, there is strong evidence that significant weight loss achieved by using lifestyle and medical methods by obese, particularly severely obese, people is modest and rarely sustained, particularly in the severely obese [5,21,22]. There are now few medications approved for weight loss with recent withdrawals associated with adverse events.

### Negative attitudes toward obesity

There are widely held community attitudes that the majority of obese individuals are responsible for their current weight. Severe obesity is too often misconstrued as a ‘cosmetic’ problem and a result of personal failure or lack of motivation.

However, this perspective ignores the very strong genetic and developmental bases to severe obesity [23] compounded by physical, emotional and societal issues. It also fails to consider the pervasive obesity-promoting effects of modern societies (the ‘obesogenic environment’) [24], where an abundant food supply, changes in food preparation, increasing sedentary behaviour and other lifestyle factors mitigate against weight control for individuals. Additionally, it ignores the emerging evidence that body weight is defended by powerful physiological mechanisms [25,26], making long-term maintenance of weight loss difficult.

In the context of treatment, negative societal attitudes have been a barrier to the provision of clinically effective, and cost-effective, health care for people with severe obesity and Type 2 diabetes [27,28]. As noted earlier, obesity is a complex, multifactorial and chronic disorder with serious adverse consequences for health, which requires a comprehensive approach to both prevention and treatment. People affected by severe obesity often struggle not only with the health and physical consequences of their chronic condition, but discrimination at work, socially and within the healthcare system.

### Why consider bariatric surgery?

Both insulin resistance and insulin secretory reserve are important in the pathogenesis of Type 2 diabetes [29], but to different extents in different people. It is very important to recognize that not all Type 2 diabetes is the same and it is currently difficult to match the different therapies available to different phenotypes often resulting to suboptimal responses to therapy.

Type 2 diabetes is a progressive disease and the usual natural history is of progressive loss of insulin secretory capacity over time and the need for intensification of therapy and polypharmacy [30]. Arresting this progression is a formidable therapeutic challenge. Treatment for Type 2 diabetes must also include active management of all cardiovascular risk factors (hypertension, dyslipidaemia, smoking and inactivity) but glycaemic control is very important—and not just for prevention of microvascular disease. Years of improved glycaemic control continue to deliver reduced risk of macrovascular disease and mortality over subsequent years [31,32].

Given the role of obesity in the aetiology of Type 2 diabetes, guidelines on its treatment provide that weight loss, with its many benefits, should be the most logical and cost-effective means of controlling Type 2 diabetes [16]. Lifestyle interventions to promote weight loss and increase physical activity should be included as an essential component of diabetes treatment regimens.

Medical therapeutic options targeting primarily glucose control are all ideally added to, and not exchanged for, lifestyle change. Unfortunately, such strategies have very limited success in controlling blood glucose levels amongst the severely obese, with many of these patients not achieving targets. A number of these medications used for treating Type 2 diabetes, including insulin, themselves can result in weight gain.

A major problem for managing Type 2 diabetes is the need for continuous monitoring and intensification of therapies by adding new agents in increasing doses over time. The American Diabetes Association (ADA) and European Association for the Study of Diabetes (EASD) consensus statement recommends that an HbA_1c_ of 7% (53 mmol/mol) is a call to action [33]. Some national guidelines, such as those from the UK's National Institute for Health and Clinical Excellence (NICE) [12], support more vigorous intensification of glycaemic therapies in the early stages of diabetes. NICE used HbA_1c_≥ 6.5% (48 mmol/mol) to increase from monotherapy, but ≥ 7% (53 mmol/mol) for increasing to triple therapies and beyond. This is very important. In one trial that randomized people with Type 2 diabetes and existing cardiovascular disease to very intensive management targeting HbA_1c_ < 6.5% (48 mmol/mol), mortality was higher in the intensive group, driven by deaths in those people who failed to show HbA_1c_ improvement as treatment was intensified [31]. This should not be taken to mean people with early Type 2 diabetes should be treated less vigorously as the legacy effect of early intervention is considerable [34].

A critical issue has been the rate at which healthcare professionals escalate therapies. Current approaches that rely on loss of glycaemic control and on intensifying lifestyle or other time-consuming measures set clinicians up for failure to achieve targets [35].

It may be possible to achieve much more in terms of complication prevention—or even possibly slowed rate of progression—if treatments are started and intensified early. There have even been suggestions of starting polypharmacy at diagnosis [36,37], but there is limited current evidence to demonstrate the efficacy of this [31].

Apart from the side-effect profiles and suboptimal deployment of existing medical diabetes therapies, there remain issues around patient engagement in many aspects of their lives. Very few clinical services routinely provide psychological support to encourage lifelong engagement in self-care.

The continuing morbidity and mortality in persons with diabetes is a sign that the answer as to the best management for Type 2 diabetes in terms of maximizing metabolic control is still elusive. Given this scenario, the option of bariatric intervention needs to be considered in appropriately selected individuals.

### Evolving concept: bariatric–metabolic surgery

The term ‘bariatric’ surgery, derived from the Greek word *baros* for weight, defines surgical procedures designed to produce substantial weight loss. Accordingly, goals of bariatric surgery originally evolved around achieving substantial sustained weight loss. In reality, weight loss is only one of the outcomes of such surgery. Bariatric surgery can be associated with substantial other health benefits, including improvement or normalization of hyperglycaemia. hyperlipidaemia, blood pressure, obstructive sleep apnoea and improved quality of life [38].

In view of the broad benefits of weight loss and the growing evidence that some bariatric procedures provide metabolic changes that cannot be explained completely by their effects on body weight alone [39], the name ‘bariatric–metabolic surgery’ is emerging as a more appropriate name.

## Bariatric surgery and Type 2 diabetes

Bariatric procedures aim to reduce weight and maintain weight loss through altering energy balance, primarily by reducing food intake and modifying the physiological changes that drive weight regain. There also appear to be independent metabolic benefits, associated with effects of incretins and possibly other hormonal or neural changes after some surgical procedures [40], in addition to weight loss. For example, rapid and sustained improvements in glycaemic control can be achieved within days of gastric bypass surgery, before any significant weight loss is evident [41,42].

A 2009 Cochrane review including patients with and without diabetes concluded that bariatric surgery resulted in greater weight loss than conventional treatment in obese class I (BMI > 30 kg/m^2^) as well as severe obesity, accompanied by improvements in co-morbidities such as Type 2 diabetes, hypertension and improvements in health-related quality of life [38].

A less rigorous systematic review and meta-analysis of 621 studies which included approximately 135 000 patients identified 103 studies reporting on the remission of the clinical and/or laboratory manifestations of diabetes [6]. Overall, 78.1% of patients had ‘remission’ of diabetes following surgery. Among patients with diabetes at baseline, 62% remained in remission more than 2 years after surgery. There were significant limitations to this review as remission was largely based on clinical reporting, not HbA_1c_ or other biochemical outcomes, and follow-up of most cohorts poorly described.

The Swedish Obese Subjects study clearly demonstrated the prevention and sustained remission of Type 2 diabetes in a group of 2037 [7] severely obese patients electing to have bariatric surgery when compared with well-matched controls at 2 and 10 years follow-up (Table 2).

**Table 2:** Two- and 10-year diabetes incidence and remission* rates from the Swedish Obese Subjects Study [7]

	Surgical	Control
2-year incident	1%	8%
10-year incident	8%	24%
2-year remission	72%	21%
10-yearremission	36%	13%

* Remission based on fasting plasma glucose < 7.0 mmol/l and not on hypoglycaemic therapy [7].

The extent of remission of Type 2 diabetes is influenced by the extent of weight loss, weight regain, duration of diabetes, the pre-surgery hypoglycaemic therapy requirements, and the choice of bariatric procedure. In addition, each patient's commitment to modifying their diet and levels of exercise within a framework of ongoing multidisciplinary care will influence outcomes.

Remarkably, there is only a sole acceptably designed prospective randomized control trial (RCT) which has investigated bariatric surgery specifically as a treatment for Type 2 diabetes [43]. It compared laparoscopic adjustable gastric banding as part of a comprehensive management programme to conventional diabetes therapy with a focus on weight loss by diet and exercise. After 2 years, remission of diabetes was significantly more common in those who had received surgery (73 vs. 13%).

### Bariatric surgery benefits beyond diabetes?

Severe obesity is associated with a large number of health problems in addition to Type 2 diabetes. A review of more than 1.4 million participants in prospective studies largely from North America, Europe and Australia show a consistent progressive rise in the mortality hazard ratios with increasing BMI [44] (Table 3). A similar analysis by the Prospective Studies Collaboration found the risk of diabetes-related death was quadrupled for morbidly obese individuals [45].

**Table 3:** Mortality hazard ratios for white non-smokers [44]

	22.5–25 kg/m^2^	30–35 kg/m^2^	35–40 kg/m^2^	40–45 kg/m^2^
White women	1.0	1.44	1.88	2.51
White men	1.0	1.44	2.06	2.93

Follow-up of participants in the Swedish Obese Subjects Study after an average of 11 years found that bariatric surgery was associated with a 29% reduction in all-cause mortality after accounting for sex, age and risk factors in this severely obese group [5]. Bariatric surgery also led to a specific reduction in cancer incidence in women [46]. Other studies have confirmed this mortality advantage when compared with community matched control subjects [9,47]. A large retrospective cohort study of almost 8000 patients who had undergone gastric bypass surgery were compared for long-term mortality with age-, sex- and BMI-matched control subjects who had applied for driver's licences (Utah, USA) [9]. The analysis reported an adjusted long-term all-cause mortality reduction of 40% in the surgical group. Specific mortality reductions in the operated group were 56% for coronary artery disease, 92% for diabetes and 60% for cancer when compared with matched controls.

It would be expected that morbidly obese patients who have bariatric surgery as a treatment primarily for Type 2 diabetes would also experience the benefits of weight loss on other aspects of their health; for example, debilitating osteoarthritis or obstructive sleep apnoea. Many studies have demonstrated major improvements in health-related quality of life following bariatric surgery using both generic and obesity-specific quality-of-life instruments [48,49].

### Is bariatric surgery cost-effective?

The costs of Type 2 diabetes are substantial. In the USA, the lifetime cost has been estimated at $US172 000 for a person diagnosed at the age of 50 years and $US305 000 if diagnosed at the age of 30 years [50]. The estimate included both the direct medical costs of diabetes and its complications and indirect costs caused by work absence, reduced productivity at work, disability and premature death. Over 60% of the medical cost was incurred within 10 years of diagnosis. Bariatric surgery for severe obesity, regardless of diabetes status, has been assessed as cost-effective [51] and, in some analyses, cost saving or dominant [52].

A literature review identified three cost-effective analyses of bariatric surgery for patients specifically with diabetes (Table 4). All three studies found bariatric surgery to be either very cost-effective or dominant as a therapy for Type 2 diabetes relative to standard therapy. Study analyses have been conservative. The finding of ‘cost-effectiveness’ indicates that health benefits are achieved at an acceptable price relative to country-specific cost-effectiveness thresholds. The ‘dominant’ result indicates that an intervention generates both cost savings and health benefits over the lifetime of the cohort. This is a rare outcome and provides the most compelling evidence for funding based on economic criteria.

**Table 4:** Cost-effectiveness of bariatric procedures in people with diabetes

Study	Type 2 diabetes status	Total costs	QALYs	Incremental cost-effectiveness ratio (ICER), Cost per QALY	Cost-effectiveness threshold/interpretation
Keating *et al*. [76], Australia, $A 2006, lifetime					$A50 000
Standard care*	Recently diagnosed	101 376	14.5	—	—
Banding surgery	Recently diagnosed	98 931	15.7	(ICER N/A) Save $2444 Generate 1.2 QALYs	Dominant
Hoerger *et al*. [77], USA, $US 2005, lifetime					$US50 000
Standard care*	Recently diagnosed	71 130	9.55	—	—
Bypass surgery	Recently diagnosed	86 655	11.76	7000	Very CE
Banding surgery	Recently diagnosed	89 029	11.12	11 000	Very CE
Standard care*	Established	79 618	7.68		
Bypass surgery	Established	99 944	9.38	12 000	Very CE
Banding surgery	Established	96 921	9.02	13 000	Very CE
Picot *et al*. [51], UK, £ 2006, 20 years					£20–30 000
Standard care*	Recently diagnosed	31 683	10.39	—	—
Banding surgery	Established	33 182	11.49	1367	Very CE

* Base case.

CE, cost-effective; QALY, quality-adjusted life-years.

In mid 2006: 1 Euro = $A1.72/£0.69/$US1.28.

It is recognized that cost-effectiveness studies have not been conducted in low- and middle-income countries where high-cost interventions for macro- and microvascular complications may not be available. However, life expectancy might indeed be improved by bariatric surgery in these settings and morbidity decreased. It is up to each health system to determine whether bariatric surgery with its support services is economically appropriate when weighed against the provision of essential medicines and other secondary prevention initiatives, such as foot care, education and retinal screening, which can be cost saving in low-income countries.

### What eligibility guidelines exist?

A number of guidelines exist on the use of bariatric surgery for the treatment of severe obesity in general, and for the treatment of Type 2 diabetes in particular. They are summarized in Table 5. Most of the existing guidelines reflect the expert recommendations of the National Institutes of Health (NIH) Consensus Development Conference Statement March 1991. The current NIH website warns that their information is dated and provided solely for historic purposes [53].

**Table 5:** National and international guidelines* for eligibility for bariatric surgery (adults)

	NIH [78] (USA)	NHMRC [59] (Australia)	NICE [12] (UK)	European [10]	ADA [63] (USA)	SIGN [79] (Scotland)
Year	1991	2003	2006	2007	2010	2010
Recommended:BMI			> 50 kg/m^2^			
Eligible (A): BMI	> 40 kg/m^2^	> 40 kg/m^2^	> 40 kg/m^2^	> 40 kg/m^2^	> 40 kg/m^2^	
Eligible (B): BMI	35–40 kg/m^2^ with one serious weight-loss-responsive co-morbidity	35–40 kg/m^2^ with one serious weight-loss-responsive co-morbidity	35–40 kg/m^2^ with disease that could improve with weight loss	35–40 kg/m^2^ with one weight-loss-responsive co-morbidity	35–40 kg/m^2^ if control of diabetes and co-morbidity is difficult	> 35 kg/m^2^ with one serious weight-loss-responsive co-morbidity
Comment	Medicare NCD 2004 removed ‘serious’ BMI 30–35 kg/m^2^	Recognized use < BMI 35 kg/m^2^		Weight loss pre-surgery does not change eligibility	BMI < 35 kg/m^2^ insufficient evidence to date	
Review	Outdated Of historic interest	Review in 3 years suggested				

* The guidelines above are qualified by the following common elements: appropriate non-surgical weight-loss measures have been tried and failed; there is the provision for, and a commitment to, long-term follow-up; and individual risk–benefit ratio needs to be evaluated.

ADA, American Diabetes Association; NCD, non-communicable disease; A, eligible BMI; B, eligible conditional BMI; NHMRC, National Health and Medical Research Council; NICE, National Institute for Health and Clinical Excellence; NIH, National Institutes of Health; SIGN, Scottish Intercollegiate Guidelines Network.

A recent Diabetes Surgery Summit of 50 international experts examined gastrointestinal surgery for the management of Type 2 diabetes. Delegates strongly endorsed that conventional gastrointestinal surgery—Roux-en-Y gastric bypass (RYGB), laparoscopic adjustable gastric band (LAGB) or bilio-pancreatic diversion (BPD)—should be considered for the treatment of Type 2 diabetes in acceptable surgical candidates with BMI > 35 kg/m^2^ who are inadequately controlled by lifestyle and medical therapy. Further trial evidence was deemed necessary for inadequately controlled Type 2 diabetes in candidates suitable for surgery with mild-to-moderate obesity (BMI 30–35 kg/m^2^) [14].

#### Recommendations for adolescents

Long-term whole-of-family lifestyle change, with high-quality medical management, is the mainstay of paediatric obesity treatment. However, the growing prevalence of severe obesity in children and adolescents demonstrates a need for additional therapy. Bariatric surgery is only considered suitable for adolescents of developmental and physical maturity. There are a range of guidelines and consensus reports that have similar recommendations.

A recent position statement was developed by the Australian and New Zealand Colleges for paediatric physicians and surgeons and the Obesity Surgery Society of Australia and New Zealand [54]. The statement recommended surgery be considered if adolescents had BMI > 40 kg/m^2^, or > 35 kg/m^2^ with severe co-morbidities (including Type 2 diabetes), were aged 15 years or more, with Tanner pubertal stage 4 or 5 and skeletal maturity, and could provide informed consent. Potential candidates should have failed a multidisciplinary programme of lifestyle ± pharmacotherapy for 6 months, and they and their family must be motivated and understand the need to participate in post-surgical therapy and follow-up. Surgery should be provided in units affiliated with teams experienced in the assessment and long-term follow-up of the metabolic and psychosocial needs of adolescent patients. Very similar eligibility criteria, with some variation in youngest age and BMI, have been listed in European and US publications [10,55].

This IDF position statement advises that only two procedures, namely Roux-en-Y gastric bypass (RYGB) and laparoscopic adjustable gastric banding (LAGB), are currently conventional bariatric surgical procedures for adolescents.

### Do procedures vary in effectiveness?

A number of bariatric surgical procedures are effective in achieving weight loss. Those that involve more extensive surgery, such as Roux-en-Y gastric bypass, generally lead to greater weight loss and more profound metabolic changes, at least initially, than less invasive, non-diversionary procedures such as laparoscopic adjustable gastric banding. Roux-en-Y gastric bypass procedures influence the gut hormonal milieu and provide an early non-weight related improvement in glycaemic control of Type 2 diabetes. It is not clear if these changes are durable or have a fundamental effect on the underlying mechanisms driving Type 2 diabetes. In the longer term, weight loss may be the key benefit. There is absolutely no evidence to support subcutaneous lipectomy (liposuction) as a treatment for Type 2 diabetes in obese patients [56].

A systematic review of the literature by Buchwald *et al*. [6] reported that diabetes remits or improves in the majority of patients after bariatric surgery. The procedures producing greater excess weight loss lead to higher remission rates (Table 6). This review, however, was limited by the quality of the available literature where follow-up varied, there was no consistent definition of remission, and biochemical measures of remission were usually not reported.

**Table 6:** Estimated weight loss and percentage of those with diabetes who remit at 2 years after conventional bariatric procedures*

	% excess BMI loss†	% remission of diabetes
Bilio-pancreatic diversion	73	95
Roux-en-Y gastric bypass	63	80
Laparoscopic adjustable gastric band	49	57

* Systematic review (Buchwald *et al*. [6]).

† Mean % on BMI in excess of 25% that is lost.

The choice of bariatric procedure is complex, requiring a careful risk–benefit analysis and acceptance of variation in regional practice and expertise. The decision must be made by severely obese patients in consultation with their bariatric surgical multidisciplinary team.

### Text box 3 Factors to consider when choosing a procedure in patients with Type 2 diabetes include

**Table:** 

• Expertise and experience in the bariatric surgical procedures
• The patient's preference when the range of risks and benefits, the importance of compliance, and the effects on eating choices and behaviours have been fully described
• The patient's general health and risk factors associated with high perioperative morbidity and mortality
• The simplicity and reversibility of a procedure
• The duration of Type 2 diabetes and the degree of apparent residual B-cell function
• The follow-up regimen for the procedure and the commitment of the patient to adhere to it

It is important to recognize that all conventional surgical procedures vary in their risks and benefits and, to date, there are few hard data that can be used to match patients to procedures.

Recommendations made by this consensus apply to currently accepted bariatric surgical procedures and do not apply to new experimental procedures or devices.

The consensus group consider that Roux-en-Y gastric bypass, laparoscopic adjustable gastric banding, bilio-pancreatic diversion (BPD) and the duodenal switch variant (BPD-SD), and sleeve gastrectomy (SG) as currently accepted procedures [57]. However, it was acknowledged that there was limited medium- or long-term data regarding sleeve gastrectomy, and there are safety, nutritional and metabolic concerns with bilio-pancreatic diversion and the duodenal switch variant. Two procedures were considered accepted procedures in adolescents—Roux-en-Y gastric bypass and laparoscopic adjustable gastric banding (see *Recommendations for adolescents* above).

### What are the risks of bariatric surgery?

The 30-day mortality associated with bariatric surgery is estimated at 0.1–0.3%, a rate similar to that for laparoscopic cholecystectomy [58] and described as ‘low’ [59]. Programme and patient factors found to be associated with increased risk are shown in Table 7. The presence of Type 2 diabetes has not been found to be associated with increased risk for bariatric surgery.

**Table 7:** Patient and programme factors associated with risk of surgery

Programme—surgical factors ‘higher risk’	Patients' factors ‘higher risk’ [80,81]
Surgeon inexperience or in learning curve for the particular procedure	Older age
Low volume centre or surgeon performing surgery occasionally	Increasing BMI
Morbidity and mortality increase with the complexity of the procedure	Male gender
Open compared with laparoscopic procedures	Hypertension
Revisional surgery	Obstructive sleep apnoea
	High risk of pulmonary thromboembolism
	Limited physical mobility

The most common complications of bariatric surgery include anastomotic and staple-line leaks (3.1%), wound infections (2.3%), pulmonary events (2.2%) and haemorrhage (1.7%). Morbidity rates are lower after laparoscopic procedures, which constitute a steadily increasing proportion of bariatric operations [60].

A new study by the US Agency for Healthcare Research and Quality reported a 21% decline in complications after bariatric surgery between 2002 and 2006 [61]. This work compared complications among > 9500 patients who underwent obesity surgery at 652 hospitals in 2001–2002 vs. 2005–2006. Complication rates fell from ∼24 to 15%, despite increases in the percentage of older and sicker operative patients. Post-surgical infection rates dropped by 58%, while other complications such as abdominal hernias, staple leakage, respiratory failure and pneumonia diminished by 29–50%. Other complications remained unchanged (ulcers, dumping, haemorrhage, wound re-opening, deep-venous thrombosis and pulmonary embolism, heart attacks and strokes) and none increased.

Early post-operative morbidity and mortality are related to the complexity of the surgery. The US Bariatric Outcomes Longitudinal Database (BOLD) reviewed over 57 000 consecutive procedures and reported one or more complication at 1-year rates of 4.6, 10.8, 14.9 and 25.7% following laparoscopic adjustable gastric band, sleeve gastrectomy, Roux-en-Y gastric bypass and bilio-pancreatic diversion, respectively [62]. Thirty-day post-surgical mortality follows a similar trend, with 0.1% for laparoscopic adjustable gastric band, 0.5% for Roux-en-Y gastric bypass and 1.1 for bilio-pancreatic diversion [58]. The US Agency for Healthcare Research and Quality reported a ninefold increase in bariatric surgery for the period 1998–2004, with a reduction in overall early mortality from 0.89 to 0.19%. Improvements have been attributed to higher hospital volumes, a move to laparoscopic surgery and an increase in banding procedures [61].

Longer-term surgical complications and need for surgical revisions are not uncommon and expected problems are usually specific to the surgical intervention.

Early detection and appropriate management of complications is very important. All those managing post-bariatric surgical patients should have a low threshold for surgical referral should a complication be suspected. Longer-term concerns, especially with Roux-en-Y gastric bypass and bilio-pancreatic diversion, include vitamin and mineral deficiencies, osteoporosis and, rarely, Wernicke's encephalopathy and severe hypoglycaemia from insulin hypersecretion [11,63]. Clinical guidelines developed by the American Association of Clinical Endocrinologists, The Obesity Society and the American Society for Metabolic and Bariatric Surgery address these important issues [11]. A summary of nutritional risk with each procedure is shown in Table 8. This does not reflect all nutritional risks. Long-term dietary advice, evaluation and supplementation are required for all procedures.

**Table 8:** A summary of more common nutritional concerns for each procedure

	LAGB	SG	RYGB	BPD	BPD-DS
Iron	+	++	+++	+++	++
Thiamine	+	++	+	+	+
Vitamin B12	+	++	+++	++	++
Folate	++	++	++	++	++
Calcium	+	++	++	+++	+++
Vitamin D	+	+	++	+++	+++
Protein	+	+	+	++	++
Fat-soluble vitamins and essential fatty acids	+	+	+	+++	+++

+, recommended daily intake (allowance) or standard multivitamin preparation likely to be sufficient.

++, significant risk of deficiency or increased requirements. Specific supplementation is appropriate especially in higher-risk groups.

+++, high risk of deficiency. Additional specific supplementation is necessary to prevent deficiency. Careful monitoring is recommended. Supplementation well in excess of daily requirements may be necessary.

BPD, bilio-pancreatic diversion; BPD-DS, bilio-pancreatic diversion with duodenal switch; LAGB, laparoscopic adjustable gastric band; RYGB, Roux-en-Y gastric bypass; SG, sleeve gastrectomy.

The risks of each procedure need to be considered in the light of potential reductions in mortality, morbidity or co-morbidity, quality of life and productivity. Realistic expectations are important and the risk–benefit ratio assessed individually for each patient, accounting for both peri-operative risk and possible long-term complications [59].

Continuing efforts are required to monitor the safety, efficacy and long-term effects of bariatric surgery. There is a range of national bariatric surgery registries and continuing long-term longitudinal studies. We encourage the expansion of national registries and acknowledge that these must be well resourced to function appropriately. Severe obesity and Type 2 diabetes are chronic conditions needing a chronic-disease approach to care.

### Components of successful bariatric surgery

There is a range of comprehensive guidelines for the use of bariatric procedures for obesity, including the UK National Institute for Health and Clinical Excellence (2006) [12], the combined American Association of Clinical Endocrinologists, The Obesity Society and the American Society for Metabolic and Bariatric Surgery guidelines (2008) [11] and European clinical guidelines (2007) [10].

### Text box 4 Considerations with respect to Type 2 diabetes and components of successful programmes include

**Table:** 

• Bariatric surgery is a component of the ongoing process of chronic disease management of Type 2 diabetes and obesity
• Bariatric surgery should be performed in high-volume centres with multidisciplinary teams that are experienced in the management of obesity and diabetes. Members of the team should have understanding across disciplines and work together with common expectations and goals. The team needs to integrate with primary care, diabetes management, nutritional and lifestyle support, and surgeon's teams with consistent messages and agreed policies
• The surgical team must have undertaken relevant supervized training and have specialist experience in types of bariatric surgery performed within the programme
• Pre-surgical assessment needs to be comprehensive, including assessment of metabolic, physical, psychological and nutritional health. Patients should have realistic expectations of the risks and benefits of surgery along with their lifelong role in lifestyle intervention, nutritional support and follow-up
• Management of diabetes and other co-morbidities should be optimized and short-term pre-operative weight loss considered to improve health and visibility at the time of surgery
• The multidisciplinary team need to understand and recognize early and long-term complications in a timely manner and know when to refer back to the surgeon or others for specific care
• Lifelong follow-up on at least an annual basis is needed for ongoing lifestyle support, and post-surgical and diabetes monitoring
• Teams should collect prospective data and measure diabetes outcomes in methods consistent with IDF recommendations
• Regular, post-operative nutritional monitoring is required, with attention to appropriate diet, monitoring of micronutrient status and individualized nutritional supplementation, support and guidance to achieve long-term weight loss and weight maintenance
• Follow-up should include a psychological evaluation, support and therapy if appropriate. Mental illness, especially depression, is common in diabetes and severe obesity
• In order to help sustain weight loss from bariatric surgery, patients must be committed to increased levels of ongoing daily physical activity
• All practices are encouraged to engage and promote national programmes of ‘centres of excellence’ or equivalent and collect prospective data through registries

### Diabetes—who to consider for surgery?

There is clear evidence that bariatric surgery is a very effective therapy for obese patients with Type 2 diabetes. The place of surgery in diabetes treatment algorithms needs to be established (see below). Currently, surgery is considered optional and, as such, in the countries with the highest bariatric surgery uptake, less than 2% of eligible patients are treated annually.

Indications for bariatric surgery typically classify those who are eligible for surgery, but a recommendation of surgical referral as best practice or prioritization has not been widely considered. Diabetes management algorithms should now include points at which bariatric surgery should be considered and points at which referral is recommended or prioritized (Table 9).

**Table 9:** Eligibility and prioritization for bariatric surgery based on failed non-surgical weight-loss therapy*, BMI, ethnicity† and disease control

BMI range	Eligible for surgery	Prioritized for surgery
< 30 kg/m^2^	No	No
30–35 kg/m^2^	Yes—conditional‡	No
35–40 kg/m^2^	Yes	Yes—conditional‡
> 40 kg/m^2^	Yes	Yes

* In all cases, patients should have failed to lose weight and sustain significant weight loss through non-surgical weight-management programmes, and have Type 2 diabetes that has not responded adequately to lifestyle measures (± metformin) with a HbA_1c_ < 53 mmol/mol (7%).

† Action points should be lowered by 2.5 BMI point levels for Asian people [74].

‡ HbA_1c_ > 58 mmol/mol (7.5%) despite fully optimized conventional therapy, especially if weight is increasing, or other weight responsive co-morbidities not achieving targets on conventional therapies. For example, blood pressure, dyslipidaemia and obstructive sleep apnoea.

In patients with Type 2 diabetes, eligibility or prioritization for surgery should consider BMI, ethnicity, associated weight-related co-morbidity, weight trajectory and the response of diabetes and co-morbidity to optimal medical therapy. Conditional eligibility or prioritization should be assessed by a team specializing in diabetes. Surgical referral implies a thorough bariatric surgical multidisciplinary team assessment of risk and benefit.

Contraindications for bariatric surgery include: current drug or alcohol abuse; uncontrolled psychiatric illness; and lack of comprehension of the risks–benefits, expected outcomes, alternatives and lifestyle changes required with bariatric surgery [11]. In addition, there are general conditions that would contraindicate elective surgery and specific conditions that substantially increase the risk of surgery, later complications or poor outcomes. These should be assessed by the surgical team.

### Integration into diabetes treatment algorithms

Existing international treatment guidelines for Type 2 diabetes provide little information or direction on the role of bariatric interventions in treatment. By contrast, the American Diabetes Association recommends that bariatric surgery be considered as a treatment option for Type 2 diabetes when the patient's BMI exceeds 35 kg/m^2^ [63]. Algorithms developed for treating Type 2 diabetes should include recommendations as to where bariatric surgery is an option and the circumstances where it should be prioritized.

Almost all severely obese patients are unsuccessful in their efforts to achieve sustained and significant weight loss and there is evidence that weight loss induced by bariatric surgery can lead to remission of hyperglycaemia in the majority of patients with diabetes [6,64]. Earlier intervention increases the likelihood of remission [65,66]. In the remaining patients, residual hyperglycaemia is easier to manage following bariatric surgery. It can therefore be argued that bariatric surgery for the severely obese with Type 2 diabetes should be considered early as an option for eligible patients, rather than being held back as a last resort.

### Equity of access to bariatric surgery

Obesity is more common in socio-economically disadvantaged people in the developed world, but the vast majority of bariatric surgery procedures in the developed world are performed in the private sector. Current access to surgical treatment for people with severe obesity and Type 2 diabetes is not equitable and discriminates against individuals who are most likely to benefit. There are particular problems in those emerging countries where rates of severe obesity are increasing rapidly and healthcare resources are extremely limited.

There will be resource implications in the short term from increasing access to bariatric surgery, but it is essential to consider not just the financial costs of the procedures and necessary follow-up, but also the potential savings from achieving improved control of Type 2 diabetes, its related metabolic and other complications and co-morbidities.

### Measuring diabetes-related outcomes

There needs to be an agreed definition of success and, on the basis of present data, the achievable goal of bariatric surgery is not cure, but remission, of the diabetes state. Improved patient health would be recognized by individualized optimization of the metabolic state, which involves normalization or improvement of the metabolic state (Text box 5).

### Text box 5 Criteria for remission or optimal metabolic state and substantial improvement

**Table:** 

Optimization of the metabolic state may be defined as:
• HbA_1c_≤ 42 mmol/mol (6%)
• no hypoglycaemia
• total cholesterol < 4 mmol/l; LDL cholesterol < 2 mmol/l
• triglycerides < 2.2 mmol/l
• blood pressure < 135/85 mmHg
• > 15% weight loss
• with reduced medication from the pre-operated state or without other medications (where medications are continued, reduced doses from pre-surgery with minimal side effects would be expected)
A substantial improvement in the metabolic state may be defined as:
• lowering of HbA_1c_ by > 20%
• LDL < 2.3 mmol/l
• blood pressure < 135/85 mmHg
with reduced medication from the pre-operated state

The above definitions, with a focus on diabetes, complement broader success measures, including substantial sustained weight loss, improved quality of life and improvement or remission of obesity-associated co-morbidity.

### Type 2 diabetes: novel procedures and devices

#### Novel bariatric–metabolic procedures

Several novel procedures have developed from elegant experiments using rodent models to examine the mechanism of action of bariatric surgery. The aim has been to enhance the non-weight-loss glycaemic control benefits of the gastrointestinal interventions. These procedures may evolve as therapy for Type 2 diabetes in those without significant weight issues. These novel procedures include duodenal–jejunal bypass (DJB) [67] and ileal interposition (IT) [68].

First described by Rubino [69], duodenal–jejunal bypass is a stomach-sparing bypass of a short portion of proximal intestine, comparable with the segment excluded in a standard Roux-en-Y gastric bypass. A number of early human clinical trials have been performed and improvements in glycaemic control have been reported, but these may be less impressive in subjects with a lower BMI [70,71].

Ileal interposition involves the surgical transposition of a small segment of ileum into the proximal intestine. Generally, short-term studies in humans have reported improved glycaemia [72,73].

These procedures remain experimental and are likely to require technical refinements before larger-scale longer-term safety and efficacy studies.

#### Novel bariatric–metabolic devices

Multiple, mostly novel, devices and techniques are being explored to utilize the gatrointestinal tract's putative mechanism for altering energy balance and for non-weight-loss effects on glucose tolerance. In general, the techniques can be divided by mode of placement into those that are upper gastrointestinal endoscopic or laparoscopic, with some combining approaches.

Endoscopically placed upper gastrointestinal devices include the simple positioning of a device in the upper gastrointestinal tract. Examples include intra-gastric balloons, which are currently available for temporary placement (usually 6 months, but repeat treatment for extending the duration of treatment beyond 2 years have been reported) and which provide 10–15% weight loss during the period of placement, plus a range of novel devices under development, which are placed in the stomach to mimic restriction, or placed in the trans-pyloric area to delay or regulate gastric emptying. Some endoscopically placed devices are physically fixed to the upper gastrointestinal tract to mimic proximal gastric restriction of the laparoscopic adjustable gastric band, while some use endoluminal impervious sleeves to bypass the gastro-duodenal upper jejunal area to mimic the Roux-en-Y gastric bypass, or bypass the duodenum and proximal jejunum to mimic the duodenal–jejunal bypass.

A range of laparoscopic procedures to place novel electronic gastric or gastro-duodenal motility stimulators, and vagal nerve-blocking devices are also under investigation. Results in humans to date have been mixed, with some devices providing inadequate weight loss and others promising results. These are considered less invasive than most conventional bariatric surgical procedures.

Whilst there is excitement in the novel medical device area, the efficacy, safety, durability and clinical utility of many of these procedures in the management of obese people with Type 2 diabetes is still to be established.

## Recommendations

### Management of diabetes (A)

**Table:** 

1. Bariatric surgery is an appropriate treatment for people with Type 2 diabetes and obesity (BMI equal to or greater than 35 kg/m^2^) not achieving recommended treatment targets with medical therapies, especially where there are other obesity-related co-morbidities. Under some circumstances people with a BMI of 30–35 kg/m^2^ should be eligible for surgery
2. It is up to each health system to determine whether bariatric surgery with its support services is economically appropriate
3. Surgery should be considered as complementary to medical therapies to reduce microvascular and cardiovascular risk
4. Patients should be assessed and managed by experienced multidisciplinary teams
5. Glycaemic control should be optimized peri-operatively and should be closely monitored after surgery
6. Ongoing and long-term nutritional supplementation and support must be provided to patients after surgery
7. Apart from conventional procedures now in use, new techniques and devices should be explored in research settings only. Conventional procedures should be standardized. Other techniques, variations and novel devices can be introduced when supported by an evidence base

### Management of diabetes (B)

**Table:** 

8. Procedure selection requires appropriate assessment of risk vs. benefit of each operation as part of the process for selecting the surgical treatment options for an individual patient
9. New bariatric procedures require robust assessment for their efficacy, safety and durability, using similar principles to those for assessing new drug therapies and having regards to the benefits and risks of established therapy
10. Regional surgical expertise, multidisciplinary team experience and documented quality outcomes are important factors in the regional choice of bariatric procedures
11. There should be a minimal accepted data set for pre-surgery and follow-up to allow audit of clinical programmes, for example:
• HbA_1c_
• fasting glucose and insulin
• BMI
• waist circumference
• retinopathy status (recent eye examination)
• nephropathy (e.g. test for microalbuminuria within previous year)
• liver functions tests
• lipid profile
• blood pressure measurement
• foot exam (recent)
• documentation of medications—(glycaemia, lipids and hypertension)
• these should be used preoperatively
• fasting C-peptide where available
• auto-antibody status, e.g. anti-GAD where available
12. All longitudinal studies should include quality of life as one of the outcomes
13. It should be recognized that a prolonged period of normalization of glycaemic control has benefit even if there is eventual relapse

### Research recommendations

**Table:** 

1. Studies are needed to establish more robust criteria than BMI for predicting benefit from surgery and define which patients benefit most from which procedures
2. Studies are needed to establish the benefit of surgery for persons with diabetes and BMI < 35 kg/m^2^
3. Studies are needed to establish whether bariatric procedures prevent or slow the progressive loss of B-cell function characteristic of Type 2 diabetes
4. Studies are required to document the course of complications after surgery to obtain evidence that surgery stabilizes and ideally improves microvascular complications
5. Studies are needed to establish the duration of the benefit of surgery
6. Studies are needed to establish the mechanisms of the success of surgery and the mechanisms associated with recurrence
7. Studies are needed to establish the long-term complications of surgery
8. New techniques should be assessed rigorously for efficacy and safety and, ideally, mechanisms, and demonstrate their equivalence or superiority to classical surgical techniques, moving to human studies after appropriate preclinical studies
9. Studies are needed to define the best regimens of diabetes management post-bariatric surgery
10. It will be important to phenotype candidates for surgery to define what will be the most appropriate bariatric procedure for persons with diabetes in different age groups, different duration of diabetes, etc.
11. Randomized controlled trials are needed to evaluate and compare different bariatric procedures for the treatment of diabetes between themselves, as well as emerging non-surgical therapies

## Conclusion

Clinically severe obesity is a complex and chronic medical condition. Bariatric surgery is an effective and cost-effective therapy for Type 2 diabetes and obesity with an acceptable safety profile. Surgery provides an appropriate treatment for people with Type 2 diabetes and obesity not achieving recommended treatment targets with medical therapies, especially when there are other major co-morbidities. National guidelines for bariatric surgery need to be developed and implemented for people with Type 2 diabetes. Bariatric surgery should be incorporated into Type 2 diabetes treatment algorithms and the establishment of national bariatric surgical registries recommended.

**Table:** 

The IDF consensus meeting was held at the IDF head office in Brussels, Belgium (5-6 December 2010). This meeting was convened by:
Professor George Alberti
Imperial College, London and Newcastle University, UK
Professor John B. Dixon
Baker IDI Heart and Diabetes Institute, Melbourne, Victoria, Australia
Professor Francesco Rubino
Weill Cornell Medical College, New York, NY, USA
Professor Paul Zimmet
Baker IDI Heart and Diabetes Institute, Melbourne, Victoria, Australia
Other attendees at the meeting were:
Professor Stephanie Amiel
King's College, London, UK
Professor Louise A. Baur
University of Sydney, Australia
Professor Nam H. Cho
Ajou University School of Medicine, Korea
Dr Bruno Geloneze
Univerity of Campinas (UNICAMP), Brazil
Professor Jan Willem Greve
Atrium Medical Center, Parkstad Heerlen, the Netherlands
Professor Linong Ji
Peking Unviersity People's Hospital, China
Dr Muffazal Lakdawala
Saifee Hospital, Mumbai, India
Professor Wei-Jei Lee
Ming-Sheng General Hospital, National Taiwan University, Taipei, Taiwan
Professor Pierre Lefebvre
International Diabetes Federation and University of Liege, Belgium
Dr Carel le Roux
Imperial College London, UK
Professor Jean-Claude Mbanya
International Diabetes Federation, Younde, Cameroon
Professor Gertrude Mingrone
Catholic University of Rome, Italy
Professor Philip R. Schauer
Cleveland Clinic Lerner College of Medicine, USA
Professor Luc Van Gaal
Antwerp University Hospital, Belgium
Dr David Whiting
International Diabetes Federation, Brussels, Belgium
Professor Bruce M. Wolfe
Oregon Health and Science University (OHSU), USA
All panel members made a substantial contribution to the meeting and subsequent formulation of the position statement
